# Supplementary material for: Genome-wide interaction study of a proxy for stress-sensitivity and its prediction of major depressive disorder
Source: PLoS One. 2018 Dec 20;13(12):e0209160. doi: 10.1371/journal.pone.0209160 (PMC6301766; doi:10.1371/journal.pone.0209160)
Supplement: S1 Supporting Information — (DOCX) [file pone.0209160.s001.docx]

**SUPPORTING INFORMATION**

This document contains supporting information for: ***Arnau-Soler et al.* Genome-wide interaction study of a proxy for stress-sensitivity and its prediction of major depressive disorder**.

DEPICT analyses 2

Polygenic profiling 3

Supplemental References 6

# DEPICT analyses

Gene sets were analysed using DEPICT (<https://github.com/perslab/depict>) [1] to (i) prioritise genes in independent loci, (ii) identify reconstituted gene sets enriched by genes selected, which may represent biologically relevant pathways and systems, and (iii) determine enriched tissue/cell types.

SNPs from meta-analyzed GWIS with stress-sensitivity (SS) effect with *p* < 2x10^-5^ (see Supplemental Figure S2) were clumped using PLINK v1.9 [2] to identify 12 independently associated “lead SNPs” (LD r2 > 0.1; physical kb threshold = 500kb; 1000 Genomes Project Phase 1 CEU, GBR, TSI genotype data [3]). Associated regions were defined by linkage disequilibrium (LD) around the 12 “lead SNPs” (LD r^2^ > 0.5; 1000 Genomes Project Phase 1 CEU, GBR, TSI genotype data) and genes were selected if they mapped within or overlapping the regions identified (genome build GRCh37). Genes within the high LD HLA locus (chr6:25000000-35000000) were removed and overlapping regions merged. If no gene was present in a region, the nearest gene was selected. 13 unique genes were finally selected. By comparing these associated regions with genome-wide randomly-selected loci and matched for gene density, DEPICT determined whether these genes share biological function, based on the hypothesis that genes truly associated with stress-sensitivity will be part of the same mechanisms underlying this trait. No significant pathway or mechanism was found at FDR < 0.05. DEPICT is based on predicted function of genes derived using the results of 77 840 microarrays from two human, one rat and one mouse Affymetrix gene expression platforms from the Omnibus (GeO) database [4], each covering expression of 19 997 genes.

# Polygenic profiling

PRS weighted by SS effect ($\hat{\beta}_{SS}$) for each individual on GS:SFHS were estimated using GWIS statistics from UKB as follows,

$${\left( i \right) PRS}_{SS}=\sum_{j=1}^{m} \hat{\beta}_{SSj}{SNP}_{j}$$

Using MDD-GWAS statistics from UKB (discovery sample), we estimated for each SNP (ii) the main additive effect on MDD and (iii) the main additive effect on EPQN, from the following additive genetic models,

$$\left( ii \right) MDD= \beta_{Di}{SNP}_{i}+COVARIATES+ \varepsilon$$

$\left( iii \right) EPQN= \beta_{Ni}{SNP}_{i}+COVARIATES+$ε

Where $i \epsilon\left\{ 1\ldots n \right\}$; n = total number of SNPs on UKB sample (n = 557 813). Using these effects, we created MDD and EPQN PRS for each individual weighting by $\beta_{D}$ (PRS_D_) and $\beta_{N}$ PRS_N_ on GS:SFHS (target sample) as follows,

$$\left( ii \right) {PRS}_{D}=\sum_{k=1}^{l} \hat{\beta}_{Dk}{SNP}_{k}$$

$$\left( iii \right) {PRS}_{N}=\sum_{p=1}^{t} \hat{\beta}_{Np}{SNP}_{p}$$

Where $k \epsilon\left\{ 1\ldots l \right\}$; l ≤ n; l = number of SNPs at best MDD prediction fit in GS:SFHS and $p \epsilon\left\{ 1\ldots t \right\}$; t ≤ n; t = number of SNPs at best EPQN prediction fit in GS:SFHS.

All PRS at best fit (i.e. PRS_SS_, PRS_D_ and PRS_N_) were combined on several general linear models to assess MDD status (case-control) prediction on GS:SFHS as follows,

null model: $MDD \sim COVARIATES$

model 1: $MDD \sim{PRS}_{SS}+COVARIATES$

model 2: $MDD \sim{PRS}_{D}+COVARIATES$

model 3: $MDD \sim{PRS}_{N}+COVARIATES$

model 4: $MDD \sim{PRS}_{D}+ {PRS}_{N}+COVARIATES$

model 5: $MDD \sim{PRS}_{D}+ {PRS}_{SS}+COVARIATES$

model 6: $MDD \sim{PRS}_{N}+ {PRS}_{SS}+COVARIATES$

full model: $MDD \sim{{PRS}_{SS}+PRS}_{D}+ {PRS}_{N}+COVARIATES$

Before determining the scores, strand-ambiguous SNPs were removed from the genotype data. SNPs present in both the discovery and target samples were clumped to obtain a set of independent SNPs in approximate linkage equilibrium (r^2^ < 0.1, within a 250kb window). PRS were generated for up to 13 *p* thresholds (< 0.001, < 0.005, < 0.01, < 0.02, < 0.03, < 0.04, < 0.05, < 0.1, < 0.2, < 0.3, < 0.4, < 0.5, <=1). Scores were standardized to a mean of 0 and a standard deviation of 1 for use in further analyses. Each score was regressed on MDD status using logistic regression models adjusted for sex, age and 20 PCs and permuted 10 000 times to assess association with MDD status. Nagelkerke’s R^2^ coefficients, a likelihood-based measure extensively used in prediction of psychiatric disorders [5, 6] reflecting the proportion of MDD risk explained by each model at the observed scale, were calculated and converted into R^2^ coefficients at the liability scale using Hong Lee’s transformation [7] available from GEAR: GEnetic Analysis Repository [8]. To assess MDD risk explained at the population level, we used prevalence of 12.2% in GS:SFHS [9] and 25.8% in UKB [10]. Significance of each PRS was assesses by likelihood ratio test. Cross-validation was performed following the same procedure above using GS:SFHS as discovery sample and UKB as target sample to predict MDD phenotype (dependent variable) under a quasi-binomial distribution after being pre-adjusted by centre, array and genotyping batch as random effects, in a general linear regression model adjusting by sex, age and 15 PCs. Finally, the analysis was replicated and cross-validated as detailed above using summary statistics from the most recent Psychiatric Genetic Consortium MDD meta-analysis and the Genetics of Personality Consortium neuroticism meta-analysis to weight PRS_D_ and PRS_N_ by the main MDD and neuroticism additive effects, respectively.

# Supplemental References

1. Pers TH, Karjalainen JM, Chan Y, Westra HJ, Wood AR, Yang J, et al. Biological interpretation of genome-wide association studies using predicted gene functions. Nat Commun. 2015;6:5890. doi: 10.1038/ncomms6890. PubMed PMID: 25597830; PubMed Central PMCID: PMCPMC4420238.

2. Purcell S, Neale B, Todd-Brown K, Thomas L, Ferreira MA, Bender D, et al. PLINK: a tool set for whole-genome association and population-based linkage analyses. Am J Hum Genet. 2007;81(3):559-75. doi: 10.1086/519795. PubMed PMID: 17701901; PubMed Central PMCID: PMCPMC1950838.

3. Genomes Project C, Abecasis GR, Auton A, Brooks LD, DePristo MA, Durbin RM, et al. An integrated map of genetic variation from 1,092 human genomes. Nature. 2012;491(7422):56-65. doi: 10.1038/nature11632. PubMed PMID: 23128226; PubMed Central PMCID: PMCPMC3498066.

4. Barrett T, Wilhite SE, Ledoux P, Evangelista C, Kim IF, Tomashevsky M, et al. NCBI GEO: archive for functional genomics data sets--update. Nucleic Acids Res. 2013;41(Database issue):D991-5. doi: 10.1093/nar/gks1193. PubMed PMID: 23193258; PubMed Central PMCID: PMCPMC3531084.

5. Cross-Disorder Group of the Psychiatric Genomics C. Identification of risk loci with shared effects on five major psychiatric disorders: a genome-wide analysis. Lancet. 2013;381(9875):1371-9. doi: 10.1016/S0140-6736(12)62129-1. PubMed PMID: 23453885; PubMed Central PMCID: PMCPMC3714010.

6. Schizophrenia Working Group of the Psychiatric Genomics C. Biological insights from 108 schizophrenia-associated genetic loci. Nature. 2014;511(7510):421-7. doi: 10.1038/nature13595. PubMed PMID: 25056061; PubMed Central PMCID: PMCPMC4112379.

7. Lee SH, Wray NR, Goddard ME, Visscher PM. Estimating missing heritability for disease from genome-wide association studies. Am J Hum Genet. 2011;88(3):294-305. doi: 10.1016/j.ajhg.2011.02.002. PubMed PMID: 21376301; PubMed Central PMCID: PMCPMC3059431.

8. Chen GB. Estimating heritability of complex traits from genome-wide association studies using IBS-based Haseman-Elston regression. Front Genet. 2014;5:107. doi: 10.3389/fgene.2014.00107. PubMed PMID: 24817879; PubMed Central PMCID: PMCPMC4012219.

9. Fernandez-Pujals AM, Adams MJ, Thomson P, McKechanie AG, Blackwood DH, Smith BH, et al. Epidemiology and Heritability of Major Depressive Disorder, Stratified by Age of Onset, Sex, and Illness Course in Generation Scotland: Scottish Family Health Study (GS:SFHS). PLoS One. 2015;10(11):e0142197. doi: 10.1371/journal.pone.0142197. PubMed PMID: 26571028; PubMed Central PMCID: PMCPMC4646689.

10. Smith DJ, Nicholl BI, Cullen B, Martin D, Ul-Haq Z, Evans J, et al. Prevalence and characteristics of probable major depression and bipolar disorder within UK biobank: cross-sectional study of 172,751 participants. PLoS One. 2013;8(11):e75362. doi: 10.1371/journal.pone.0075362. PubMed PMID: 24282498; PubMed Central PMCID: PMCPMC3839907.
