# Supplementary figures and images for: Genome-wide interaction study of a proxy for stress-sensitivity and its prediction of major depressive disorder
Source: PLoS One. 2018 Dec 20;13(12):e0209160. doi: 10.1371/journal.pone.0209160 (PMC6301766; doi:10.1371/journal.pone.0209160)

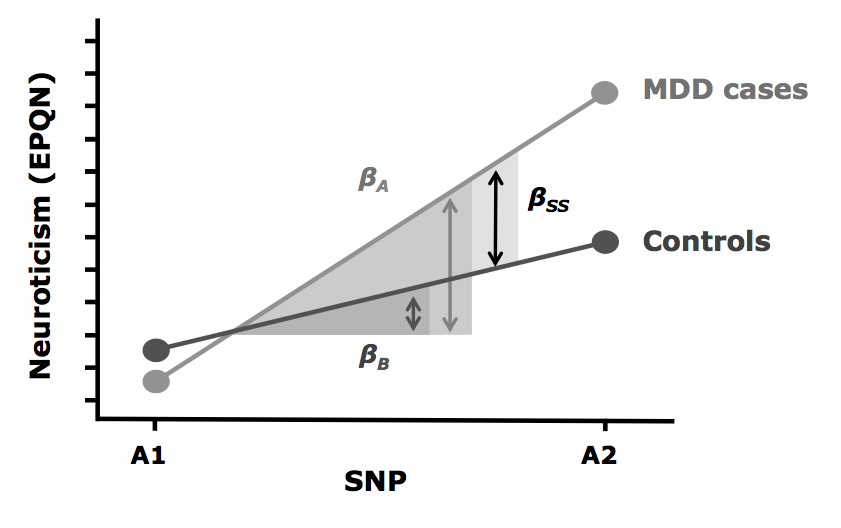

Supplement: S1 Fig — Genetic stress-sensitivity effect on MDD (βSS) is defined as the difference between the regression coefficient in MDD cases (βA) and the regression coefficient in controls (βB) from linear models regressed on EPQN, adjusted by covariates. A1: allele 1. A2 allele 2. (TIFF) [file pone.0209160.s005.tiff]

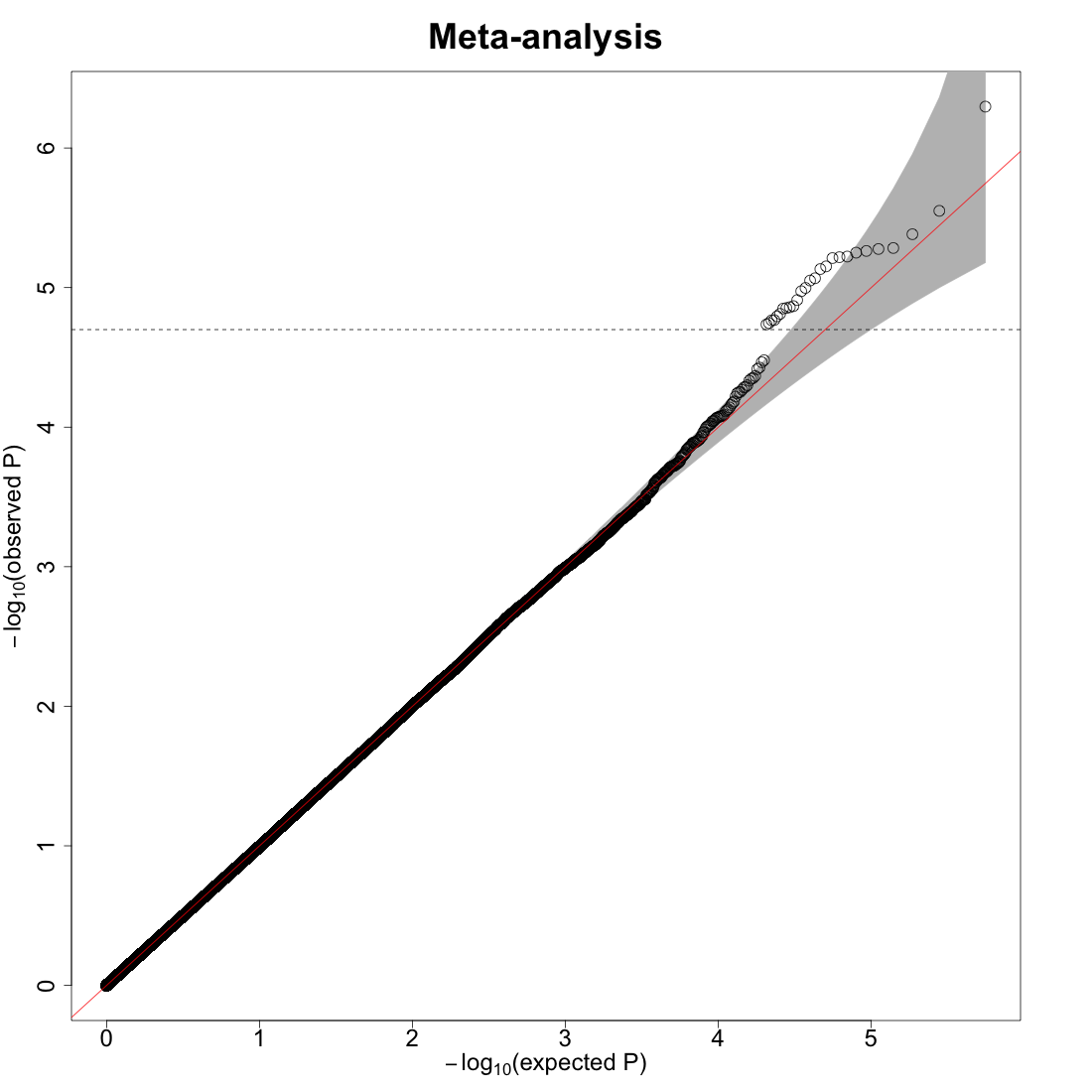

Supplement: S2 Fig — QQ plot of GWIS from sample size weighted meta-analysis (λ = 0.997; s.e. = 1.05x10^-5^). All SNPs wit p < 2x10^-5^, p threshold (dot line) where some SNPs start to deviate from null distribution going outside 95% confidence intervals (grey shadow), were selected to perform DEPICT analyses to assess pathway and functional genomic analyses. 27 top variants from 12 independent loci were selected. (TIFF) [file pone.0209160.s006.tiff]

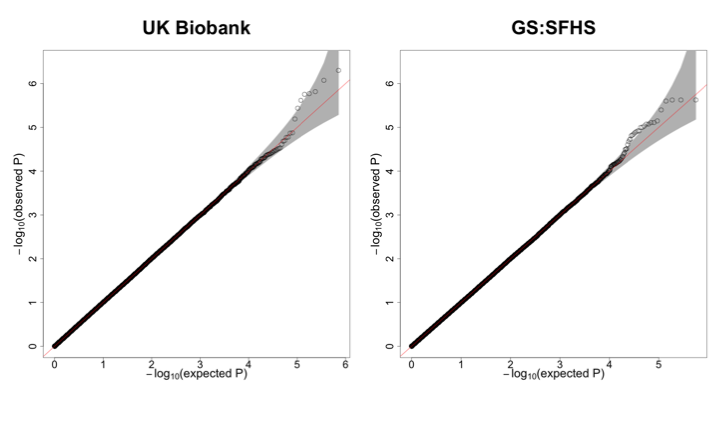

Supplement: S3 Fig — QQ plots of GWIS from (A) UKB (λ = 1.014; s.e. = 1.027x10^-5^), (B) GS:SFHS (λ = 0.997; s.e. = 7.989x10^-6^). The 95% confidence interval is shaded in grey. (TIFF) [file pone.0209160.s007.tiff]

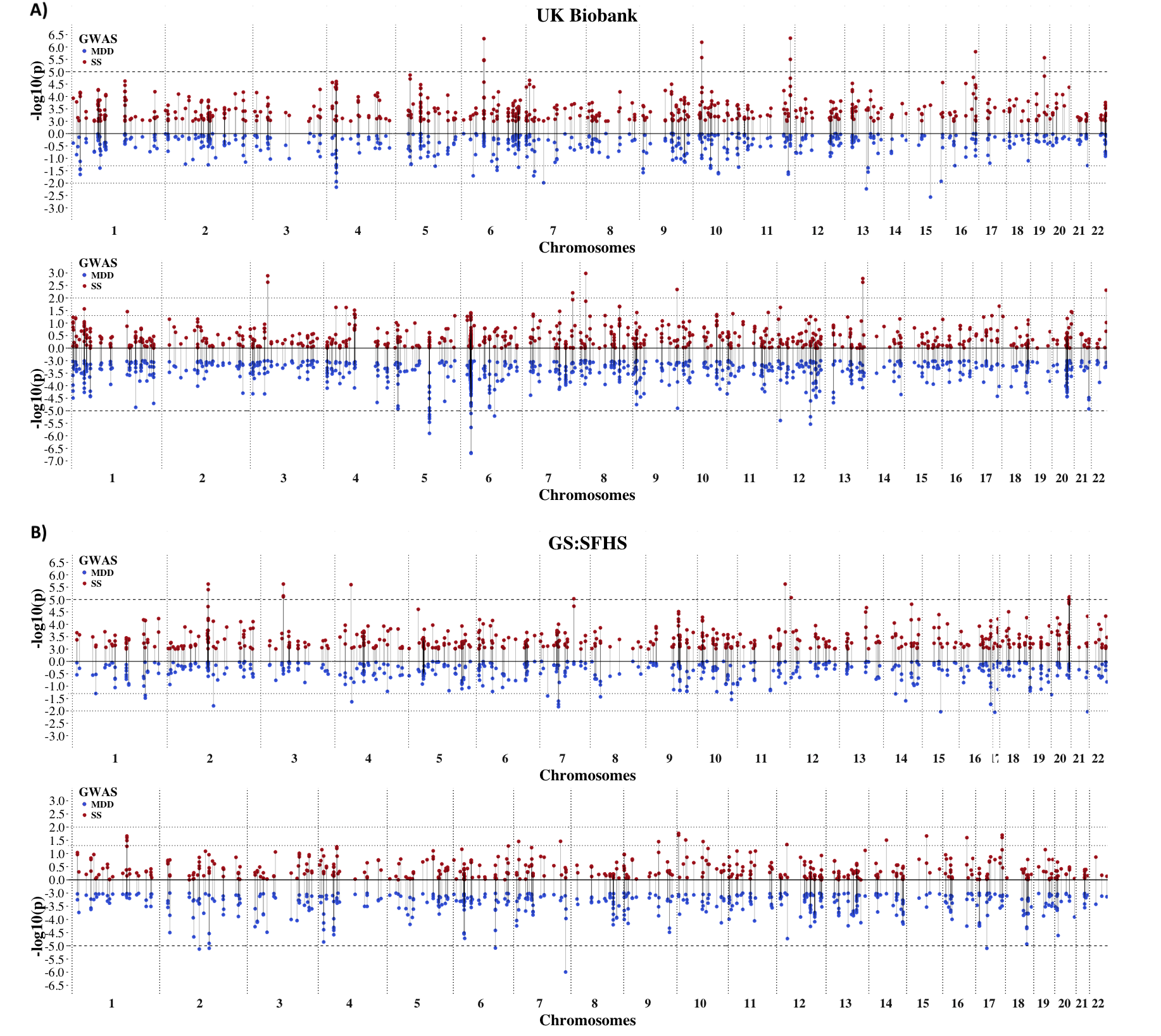

Supplement: S4 Fig — Miami plots showing comparison between association profile between SS and MDD main additive effects. Miami plots from (A) UKB filtering for SS p values (top) and MDD p values (bottom), (B) GS:SFHS filtering for SS p values (top) and MDD p values (bottom). Filter at p = 1x10^-3^. The x-axis is base-paired chromosomal position and y-axis is the significance (-log10 p) of association with (up; red dots) SS effect and (down; blue dots) MDD. Dot line: genome-wide suggestive threshold (p = 1x10^-5^) at the filtered effect; dashes lines: p value = 0.01 and 0.05 at compared effect. (TIFF) [file pone.0209160.s008.tiff]

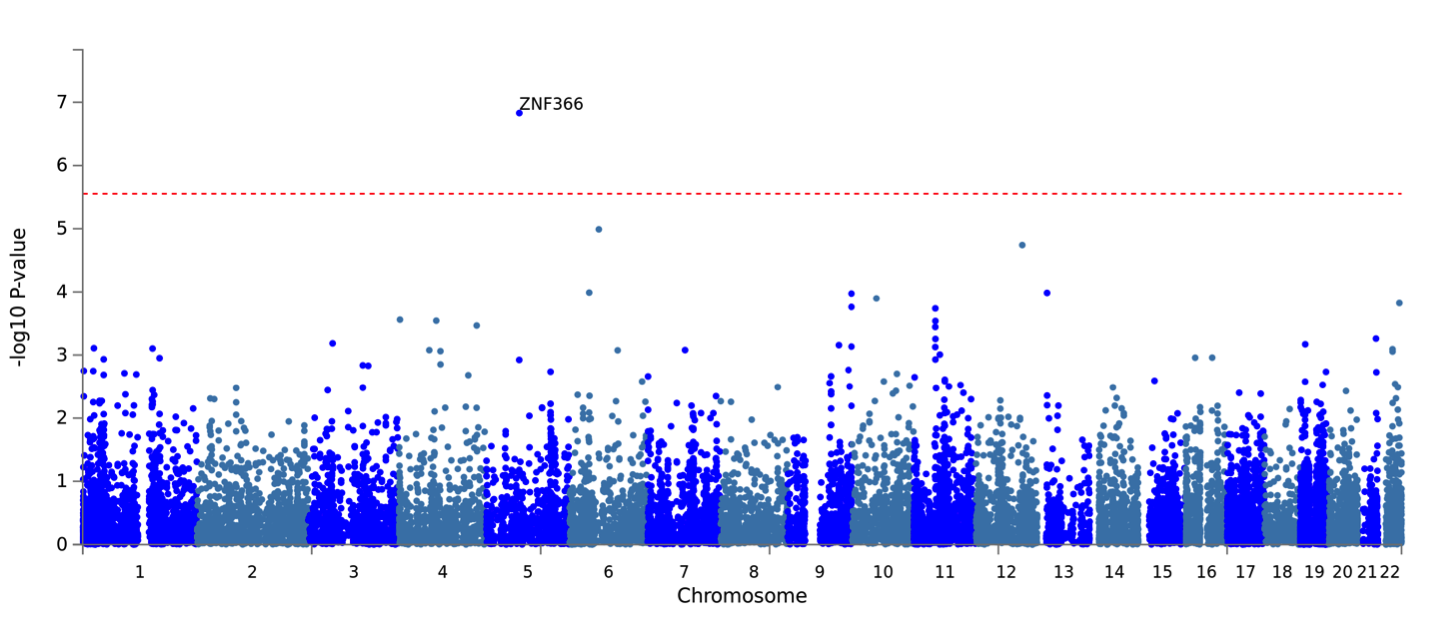

Supplement: S5 Fig — Manhattan plot showing gene-based association of stress-sensitivity. The x-axis is base-paired chromosomal position and y-axis is the significance (-log10 p value) of association with SS effect. Genome-wide significance threshold showed by red dashed line was defined at p = 0.05/17,931 = 2.79x10^-6^. (TIFF) [file pone.0209160.s009.tiff]

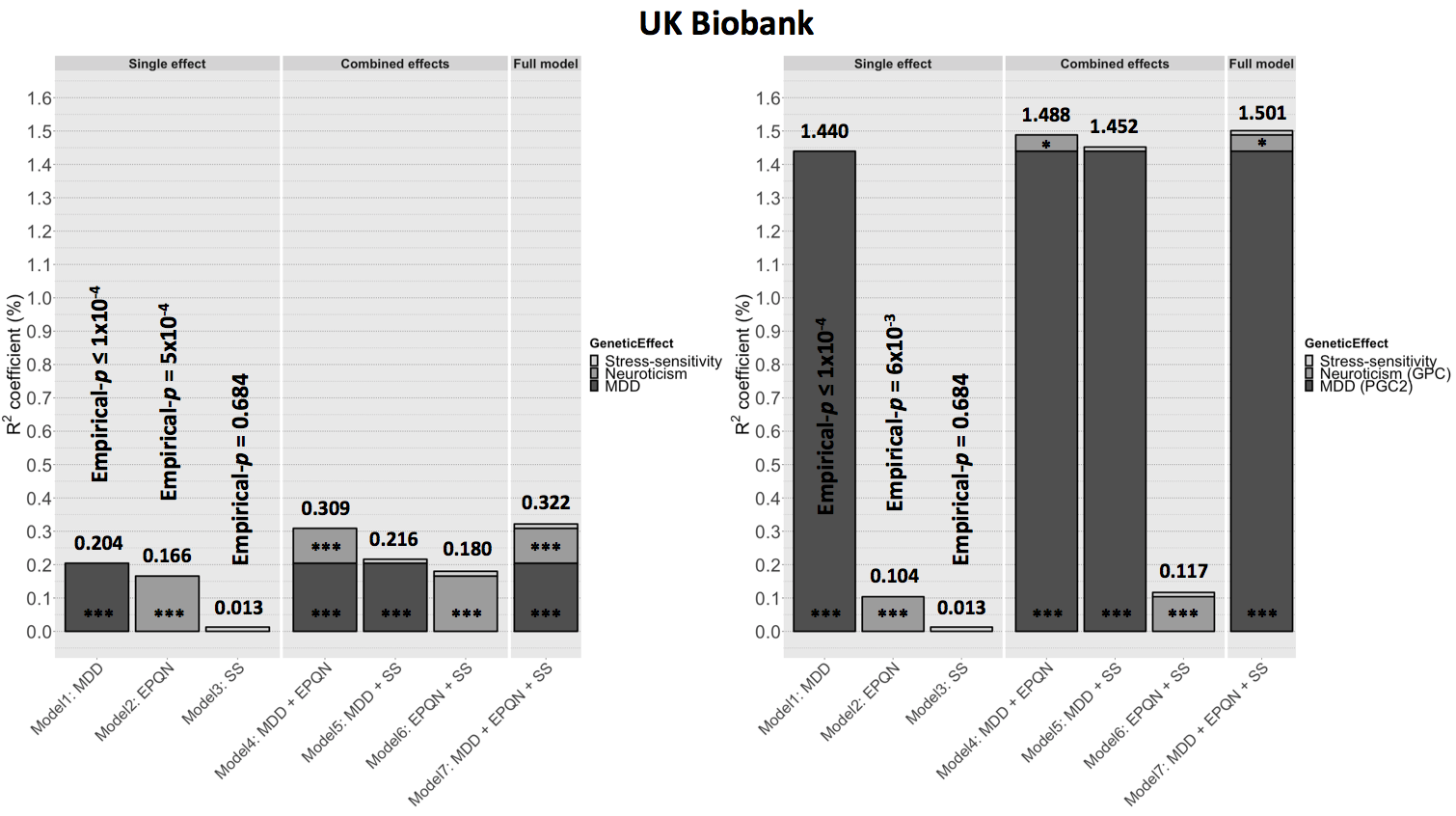

Supplement: S6 Fig — MDD risk explained (R2 coefficient (%); top bar values) on the liability scale by each PRS in UKB; weighted by GWAS main additive and GWIS stress-sensitivity effects independently and combined. (A) Using summary statistics from GS:SFHS as discovery sample. (B) Replication fitting PRSD and PRSN using summary statistics from worldwide consortiums (i.e. PGC & GPC). Significance codes: p values *** < 0.001 < ** < 0.01 < * < 0.05; derived from likelihood ratio tests. SS stands for stress-sensitivity. (TIFF) [file pone.0209160.s010.tiff]
